# Osteosarcoma of the Mandible in a Patient with Florid Cemento-Osseous Dysplasia and Li–Fraumeni Syndrome: A Rare Coincidence

**DOI:** 10.1007/s12105-020-01223-2

**Published:** 2020-09-21

**Authors:** Simon Haefliger, Dorothee Harder, Michal Kovac, Karin Linkeschova, Harald Eufinger, Daniel Baumhoer

**Affiliations:** 1 Bone Tumor Reference Center, Institute of Medical Genetics and Pathology, University Hospital of Basel, University of Basel, Schönbeinstrasse 40, 4031 Basel, Switzerland; 2 Department of Radiology, University Hospital Basel, University of Basel, Basel, Switzerland; 3 Department of Oral and Maxillofacial Plastic Surgery, Knappschaftskrankenhaus Recklinghausen, Klinikum Vest, Recklinghausen, Germany

**Keywords:** Osteosarcoma of the mandible, Li–Fraumeni syndrome, Cemento-osseous dysplasia

## Abstract

Cemento-osseous dysplasia (COD) is the most common benign fibro-osseous lesion of the jaws and generally considered non-neoplastic and self-limited. Here, we present a 30-year old female who noticed a bilateral swelling of her posterior mandible with irregular periapical mineralization and incomplete root resorption on panoramic radiographs. A biopsy revealed florid COD and no further treatment was initiated. 9 years later, she presented with a progressive expansion of her left posterior mandible after being treated for bilateral breast cancer 4 and 8 years before. CT scans showed expansile and densely mineralized lesions in all four quadrants with the left posterior mandible showing a focal penetration of the buccal cortical bone. Biopsies revealed an osteoblastic high-grade osteosarcoma in the left and a COD in the right mandible, notably with cellular atypia in the spindle cell component. The patient underwent segmental resection of the left mandible with clear margins and adjuvant chemotherapy. Subsequent genetic testing identified a heterozygous germline *TP53* mutation (p.V173G) which confirmed the clinically suspected Li–Fraumeni syndrome (LFS). 3 years after the resection, the patient is free of disease and the other foci of COD remained stable in size on follow-up imaging analyses. Our case illustrates LFS-related osteosarcoma developing within florid COD. Given the rarity of this coincidence, a causative relation between the two lesions seems unlikely but in patients with tumor predisposition syndromes it might be advisable to closely monitor even benign lesions like COD.

## Introduction

Fibro-osseous lesions of the jaws comprise cemento-osseous dysplasia (COD), ossifying fibroma (OF) and craniofacial fibrous dysplasia (FD) which can present histologically similar but show distinct clinical and imaging features. The most common member of this group is COD which has a predilection for middle-aged women of African descent. It is generally considered a non-neoplastic and self-limited, tumor-like lesion. Three subtypes can be distinguished depending on anatomic distribution: periapical COD affects the apical areas of the mandibular incisors and can be multifocal, focal COD is centered around a solitary tooth excluding the mandibular incisors and florid COD shows multiquadrant involvement. On imaging, lesions initially present lytic but progressively mineralize over time from the center to the periphery leaving a sharply delineated, dense sclerosis after full maturation [1–3]. Typically, COD is not expansile and asymptomatic, can be diagnosed radiologically and does not require bioptic confirmation or specific treatment. Some authors even consider biopsy to be contraindicated since it can result in persistent local infection and a complicated clinical course [4]. Histologically, all subtypes show a monomorphic spindle cell stroma containing an immature matrix formation that consists of woven bone and hypocellular cementum-like material that progressively coalesces to form larger, ginger root-like masses [5, 6]. OF is an odontogenic neoplasm that presents as a solitary, well delineated and expansile mass, usually with a lytic rim. Histologically, it is composed of a variable mixture of monomorphic fibroblastic spindle cells and immature bone trabeculae as well as cementum-like material. Osteoblastic rimming is a prominent feature in OF (in contrast to COD and FD) [7]. Fibrous dysplasia usually shows a painless swelling of bone with indistinct borders and ground-glass appearance, sometimes involving also the adjacent bones. The histology consists of a mature fibrous stroma encompassing an immature woven bone formation, often with a peculiar curvilinear architecture [8]. Whereas FD is known to be caused by a postzygotic activating mutation in the *GNAS* gene and to rarely undergo malignant transformation, the molecular pathogenesis for COD and OF is largely unknown and both lesions are not considered to represent precursor lesions of osteosarcoma or other malignant tumors of bone. In the case presented here, florid COD has been known for 9 years before an osteosarcoma in the context of Li–Fraumeni syndrome developed directly within the COD.

## Case Presentation

**Fig. 1:**
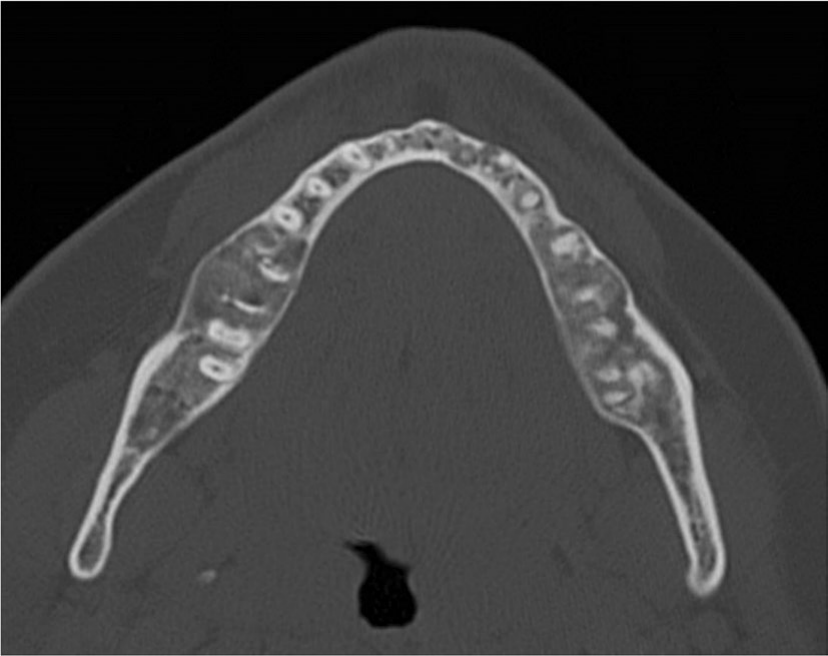
Initial axial CT scan of the jaw. Axial section of a CT scan of the mandible showing bilateral, slightly expansile and mineralized lesions without signs of aggressive behaviour

**Fig. 2:**
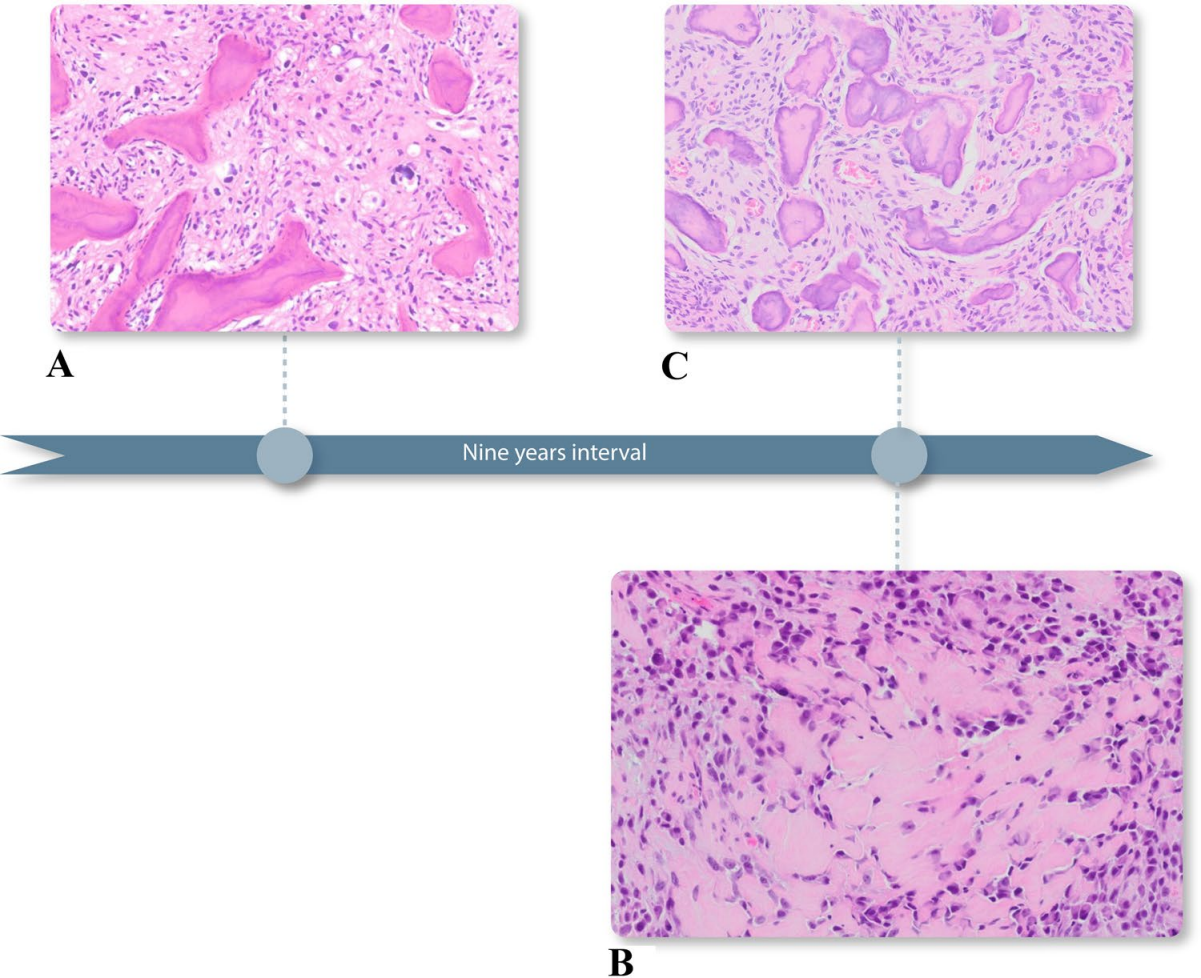
Time sequence of the different biopsies. Initial biopsy from the right mandible showing a cellular spindle cell stroma intermixed with immature woven bone and cementum-like material. Focal cytological atypia with enlarged and hyperchromatic nuclei can be observed (**a **, HE stain, ×100). Biopsy of the left mandible, 9 years later, with atypical cells producing immature bone, in line with osteoblastic high-grade osteosarcoma (**b**, HE stain, ×200). Subsequent biopsy of the right mandible showing similar findings compared with the biopsy performed 9 years before (**c**, HE stain, ×100)

A 30-year old Caucasian female presented with bilateral and painful mucosal swellings in her mandible for two months. A panoramic radiograph and a CT scan revealed slightly expansile and mineralized lesions in the posterior aspect of all four quadrants as well as perifocal root resorptions in the lower jaw (Fig. 1). There was no periosteal reaction or other signs of locally aggressive behavior. A biopsy from the right mandible consisted of a cellular spindle cell stroma encompassing immature woven bone and cementum-like material (Fig. 2a). While the stroma was mainly composed of monomorphic, fibroblast-like cells, focal cytologic atypia with enlarged and hyperchromatic nuclei was noted. Mutational analysis of the *GNAS* gene was negative and the diagnosis of florid COD was made despite the unusual root resorption and cellular atypia. Since the biopsy was small, the possibility of a perifocal inflammation causing the resorption was discussed but could not be further clarified.

**Fig. 3:**
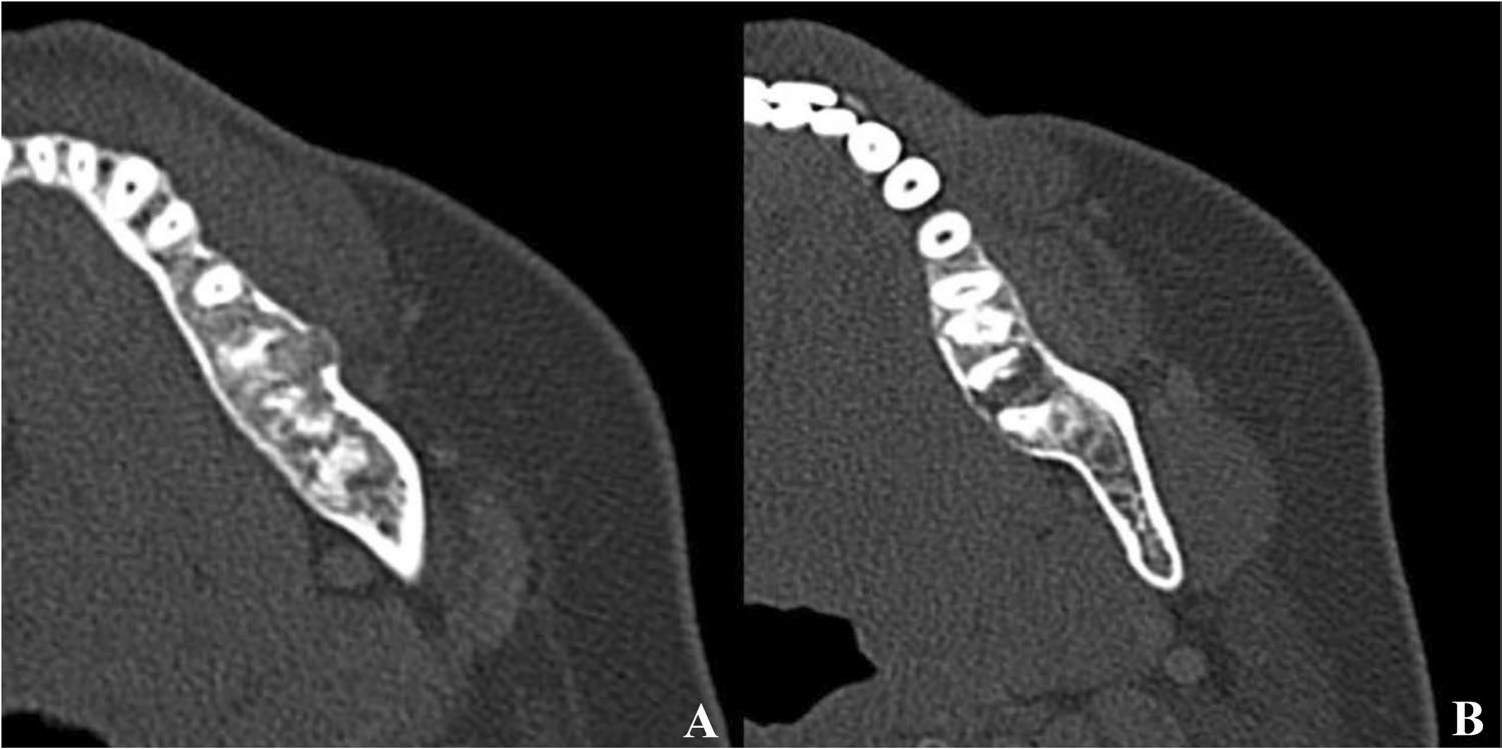
Follow up axial CT scan of the jaw. Follow-up CT scan showing progression of the known lesion of the left mandible with new and cortical penetration (**a**) as well as prominent lytic areas and progressive resorption of the adjacent dental roots (**b**)

**Fig. 4:**
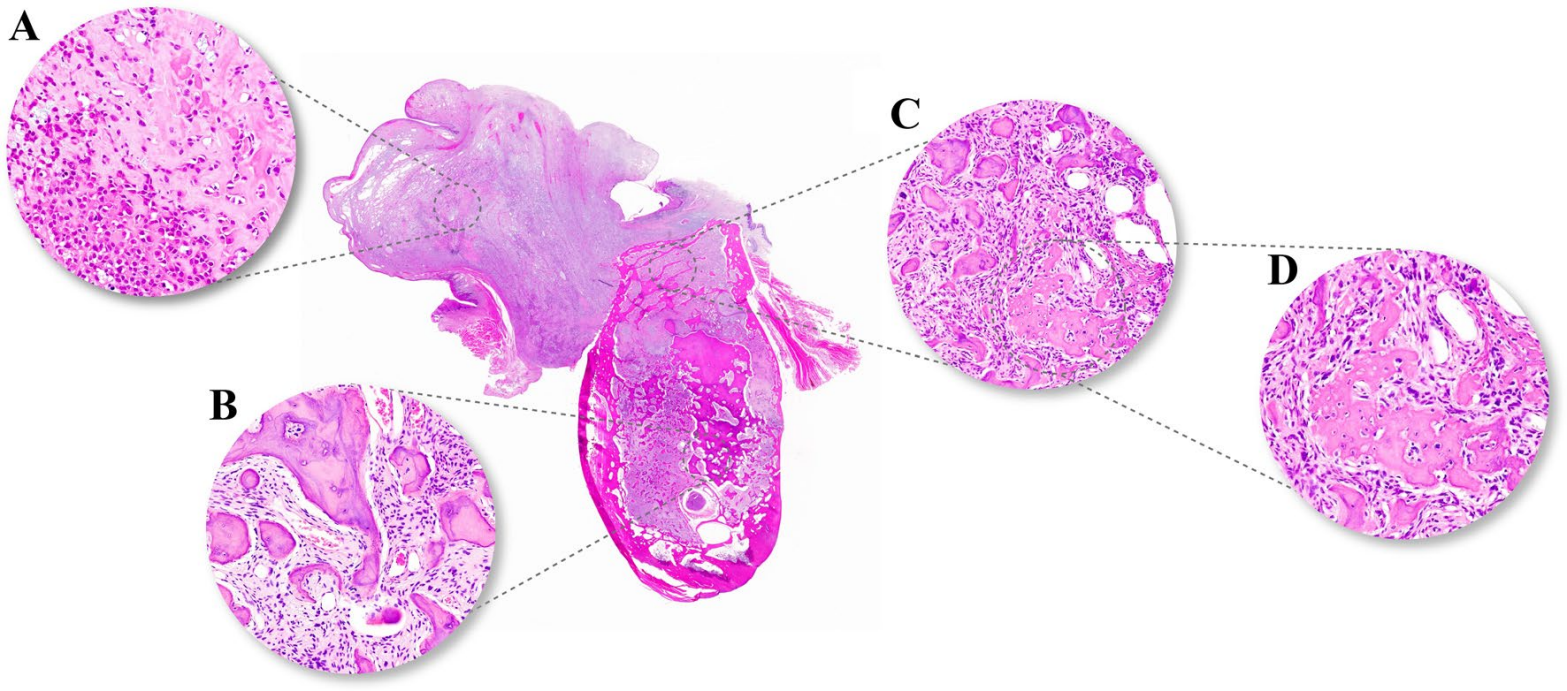
Surgical specimen. A coronal section through the mandible shows an osteosarcoma with cortical penetration and infiltration into the mucosa (**a**, HE stain, ×200). Beneath the tumor, residual areas of COD in close relation to the inferior alveolar nerve can be seen (**b**, HE stain, ×200). In the center of the specimen, the close association between COD and osteosarcoma is highlighted, as areas of COD are infiltrated by immature and neoplastic bone formation of the osteosarcoma (**c**, HE stain, ×200; **d**, HE stain, ×400)

The patient did not return until 9 years later when she reported about an increasing swelling and pain of her left posterior mandible. In the meantime, she had been treated for bilateral breast cancer 8 and 4 years before (pT1a pN0 G2 and pT2 pN0 G2). CT scans confirmed the known densely mineralized lesions in all four quadrants with the left posterior mandible appearing more aggressively affected and showing new and focal cortical penetration on the buccal aspect as well as enlarged lytic areas with progressive root resorption (Fig. 3). A biopsy was performed and revealed highly atypical cells producing immature and lace-like bone with extensive necrosis diagnostic for osteoblastic high-grade osteosarcoma (Fig. 2b). Due to the unusual histology of the right side 9 years ago, another biopsy was obtained here and again showed COD with focal atypia (Fig. 3c). A tumor staging did not reveal systemic spread and the patient underwent segmental resection of the left mandible where the diagnosis of osteosarcoma was confirmed. Notably, areas of COD were situated directly next to the osteosarcoma that penetrated through the cortex and protruded the mucosal surface (Fig. 4a–d). The resection margins were negative but due to the high-grade differentiation, adjuvant chemotherapy (nine cycles of cisplatin, doxorubicin and ifosfamide) was administered which the patient tolerated well.

Given the exceptional clinical course and the history of bilateral breast cancer, we recommended genetic counseling which the patient agreed to after finishing chemotherapy. Indeed, a heterozygous point mutation in the *TP53* gene was identified (c.518T > G; p.V173G) and the diagnosis of Li–Fraumeni syndrome was established. To better understand the genetic pathogenesis, we carried out copy number analyses (OncoScan assay, Thermo Fisher Scientific) on both biopsies from the right side and on two foci of the resection specimen from the left mandible, one from the COD and one from the osteosarcoma. Interestingly, all copy number profiles of COD were flat except for a focal loss of 17pter indicating loss of heterozygosity (LOH). Conversely, the copy number profile of the osteosarcoma was complex and showed chromoplexy with subsequent amplifications of 5p, 8pter, 12, and 19p as well as deletions of 5q, 6, 7, 10p, 13, and 22. Additional gene panel sequencing (Oncomine Comprehensive assay, Illumina) of both biopsies of the right side revealed the p.V173G *TP53* mutation as the only pathogenic mutation. Notably, the allelic frequency was 86% already in the initial biopsy supporting an LOH with loss of the wild-type allele also on the right side.

Three years after completing chemotherapy the patient is free of disease and has not developed another malignant tumor either. The COD is monitored closely with particular attention on the right mandible but has not shown any increase in size or clinical symptoms.

## Discussion

Conventional osteosarcoma is the most common primary malignant tumor of bone. It usually affects the metaphyses of long bones in children and adolescents but the jaws are the fourth most common site involved. Patients with gnathic osteosarcoma are usually one to two decades older than patients with peripheral osteosarcoma and have a more favorable outcome because metastatic spread occurs less often and later in the course of the disease [9, 10]. Most osteosarcomas arise without a known cause or precondition and although the precise mechanisms are still unclear, chromoanagenic events resulting in heterogeneous and complex chromosomal aberrations are the hallmarks of the disease. Probably caused by mitotic segregation errors these cataclysmic events usually lead to apoptosis of the affected cell but exceptionally promote tumor development. Since *TP53* is a major tumor suppressor gene, its inactivation likely increases the probability of cells to circumvent apoptosis and survive chromoanagenesis which is why patients with Li–Fraumeni syndrome are known to be at higher risk to develop malignant tumors including osteosarcoma.

In the case presented here, retrospective sequencing of a biopsy of the right mandible 9 years before osteosarcoma development on the contralateral side, identified the p.V173G *TP53* mutation with a high allelic frequency of 86%. Given the monoallelic nature of the germ line mutation found, this indicates a loss of the wild-type allele (LOH) and thus complete inactivation of *TP53*. Fortunately, the COD on the right side still remains stable 12 years after the initial biopsy and does not display any signs of dynamics in size or clinical symptoms. Nevertheless, the last biopsy 3 years ago confirmed cellular atypia in the spindle cell component similar to the first biopsy and a lack of maturation, underlining the need for a continuous and thorough monitoring of the COD.

Florid COD and Li–Fraumeni syndrome are both rare conditions and to the best of our knowledge, this is the first reported case of a patient suffering from both. The rarity of this co-incidence strongly argues against a causative relation but it may be speculated that both conditions combined generated a particularly dangerous environment for tumor development. LFS is well known for its increased risk of osteosarcoma and there are few case reports describing tumor development in the context of florid COD [11–14].

To conclude, we present a unique and first case of osteosarcoma development in a patient with Li–Fraumeni syndrome and florid cemento-osseous dysplasia. Although COD generally does not require close monitoring, a closer surveillance should be considered in patients with concomitant tumor predisposition syndromes.
